# The Role of Microglia and Matrix Metalloproteinases Involvement in Neuroinflammation and Gliomas

**DOI:** 10.1155/2013/914104

**Published:** 2013-08-14

**Authors:** Helen Könnecke, Ingo Bechmann

**Affiliations:** ^1^Clinic of Neurology, University of Zurich, Frauenklinikstrasse 26, 8091 Zurich, Switzerland; ^2^Institute of Anatomy, University of Leipzig, Liebigstrasse 13, 04103 Leipzig, Germany

## Abstract

Matrix metalloproteinases (MMPs) are involved in the pathogenesis of neuroinflammatory diseases (such as multiple sclerosis) as well as in the expansion of malignant gliomas because they facilitate penetration of anatomical barriers (such as the glia limitans) and migration within the neuropil. This review elucidates pathomechanisms and summarizes the current knowledge of the involvement of MMPs in neuroinflammation and glioma, invasion highlighting microglia as major sources of MMPs. The induction of expression, suppression, and multiple pathways of function of MMPs in these scenarios will also be discussed. Understanding the induction and action of MMPs might provide valuable information and reveal attractive targets for future therapeutic strategies.

## 1. Barriers from Blood to Brain

Influx of inflammatory cells into the neuropil is a hallmark of neuroinflammation (e.g., in multiple sclerosis (MS) [1], and respective mechanisms have been studied extensively in experimental autoimmune encephalomyelitis (EAE), an animal model for multiple sclerosis. Initially, leukocytes migrate across vascular walls and accumulate in the perivascular space. This perivascular “cuffing” [2], however, is only the first step in neuroinflammation because immune cells need to pass the glia limitans and its basement membrane to reach the parenchyma proper in a second, differentially regulated step [3]. While the endothelium does not provide an insurmountable barrier for activated T and B cells under certain (experimental) conditions [4, 5], the glia limitans and the parenchymal basal lamina represent more strictly regulated, secondary barriers [3]. Importantly, clinical symptoms only occur after the penetration of the parenchymal basal lamina (BM), which is formed by a variety of organized extracellular matrix (ECM) components build by astrocytic endfeet of the glia limitans. 

There is strong evidence that inducible proteases, known as matrix metalloproteinases (MMPs), are involved in the second step of neuroinflammation [3, 6–10]. The unique features of different, highly specialized, basal laminae rely on their major constituents: collagen IV and laminin predominant are whereas collagen type V, proteoglycans, and glycoproteins are additional constituents [11]. Collagen types IV and V are, unlike other collagens, structurally organized in a nonfibrillar, multilayer network that is resistant to nonspecific proteolytic degradation. Noteworthy is the existence of different laminin isoforms in the specialized basement membrane (BM) of the vessel and the BM of the glia limitans. While the vascular BM exhibits laminin 8 and laminin 10, the BM of the glia limitans is characterized by laminin 1 and laminin 2 [12]. Dystroglycan is a transmembrane receptor that anchors astrocyte endfeet to the parenchymal BM [13–15] via high-affinity interactions with laminin 1 and 2. Dystroglycan was identified as a specific substrate of MMP-2 and MMP-9 [16]. Thus MMPs, secreted by juxtavascular microglia, might control the ECM composition, and as a consequence MMPs are involved in the integrity and function of the glia limitans.

## 2. The MMP Family

The MMPs are a family of zinc containing endoproteinases that share structural domains but differ in substrate specificity, cellular sources, and inducibility. The major function is the degradation and remodeling of all components of the ECM. As a group of more than 20 structurally related enzymes, they can be divided according to their substrate affinity profile: gelatinases (MMP-2 and -9), interstitial collagenases (MMP-1, -8, and -13), broad-specific stromelysins (MMP-7 and -13), and other variants (see Table 1) [17]. Together, the MMP substrate repertoire includes the extracellular matrix components, fibrillar collagens, elastin as well as matrix proteoglycan core proteins, and furthermore an expanding range of nonmatrix substrates [18, 19]. MMPs are synthesized in an inactive proform that is activated extracellularly by proteolytic cleavage under the regulation of several inflammatory mediators, including cytokines, chemokines, free radicals and steroids [20, 21]. Moreover certain MMPs are able to activate others; for example, MMP-12 was shown to activate MMP-2 and MMP-3, thereby leading to an exacerbation of proteolytic processes [22].

**Figure 1:**
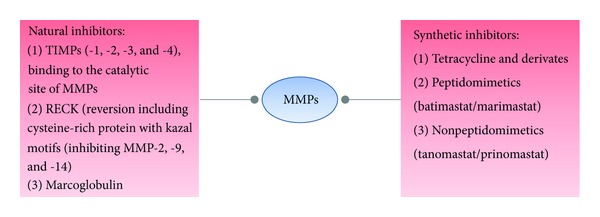
MMP inhibition is possible by targeted natural as well as synthetic inhibitors. References: Visse and Nagase [18]; Oh et al. [201]; Coussens et al. [202]; Overall and López-Otín [203].

As proteolytic enzymes, MMPs have important roles in development and physiology. They are thus linked to physiological activities in the CNS, such as myelin formation, axonal growth, angiogenesis, and regeneration [23, 24]. In general, a deviant expression or overproduction of these MMPs leads to tissue destruction, and may contribute to brain pathologies such as Alzheimer's disease, ischemia, malignant glioma, and Parkinson's disease [25–29], when not counterbalanced by their physiological inhibitors, the tissue inhibitors of MMPs, TIMPs [18] (see Figure 1). Usually MMPs are under strict control at various levels: gene transcription, synthesis, secretion, activation, inhibition and glycosylation. Therefore, normal adult CNS contains low levels of most MMP members [30], in contrast to various neurological disorders of the CNS in which several MMPs are significantly upregulated [31]. 

The upregulated MMPs in the CNS have several potentially detrimental roles, including the promotion of neuroinflammation, disruption of the blood brain barrier (BBB) [20, 32], demyelination, and damage to axons and neurons (especially MMP-1 and MMP-2) [33]. MMPs also participate in the inflammatory cascade itself by actions on inflammatory mediators and their receptors [34, 35]. Thereby, several MMPs may act in concert in a so called *MMP cascade* [13]. Moreover, MMPs may contribute indirectly to the expansion of the inflammatory response and tissue damage by generating antigens through the breakdown of myelin or by conversion of inactive membrane bound TNF-*α* into the active myelinotoxic form [36]. Similar molecules (e.g., TNF receptors, L-selectin, TGF-*β* and FAS ligand) may, due to the action of MMPs, undergo analogous processes [37]. The definite sources of the activated MMPs are still to be determined: invading T cells may release proinflammatory cytokines that activate glia cells, which are in control of the expression, secretion and balance between MMPs, as well as the secretion of their natural and specific inhibitors (TIMPS). 

## 3. Microglia in Inflammation

**Figure 2:**
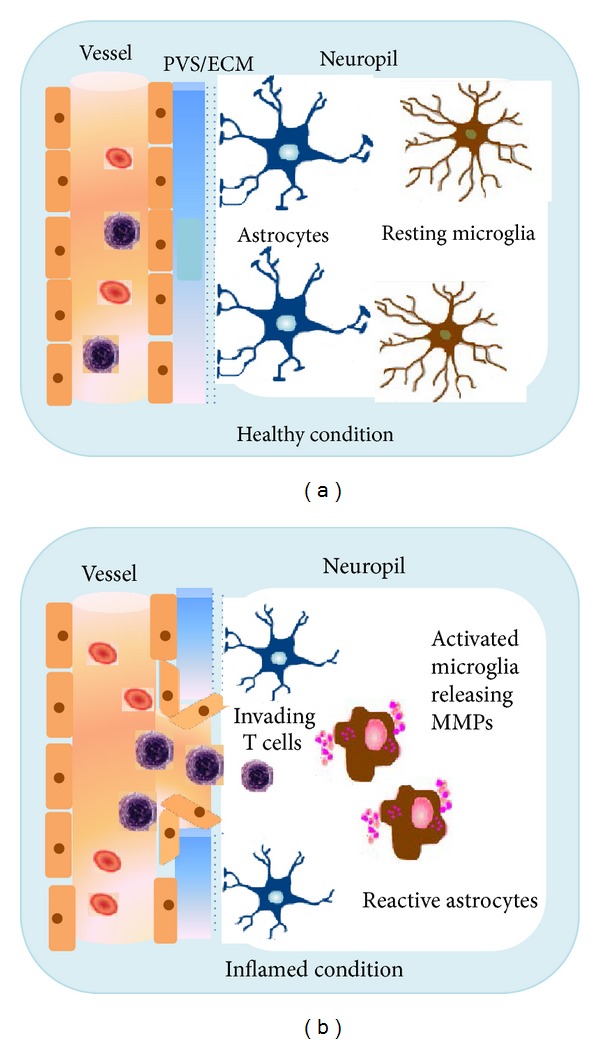
The blood brain barrier (BBB) in healthy and inflamed condition. (a) Vessel, endothelium, extracellular matrix (ECM), and glia limitans are intact. Microglia cells are in a resting state. (b) In the inflammed CNS breakdown of the BBB takes places. The glia limitans is opened, and astrocytic endfeet are drawn away. Reactive microglia secrete MMPs facilitating the opening of the BBB. Invading T cells migrate from the vessel via the perivascular space (PVS) into the neuropil.

The primary immune effector cells of the brain are microglia, which are activated in response to brain injury or inflammatory conditions. Most likely, they play a pivotal role during onset, maintenance, relapse and progression of an inflammatory state. In the course of activation, they do not only release neurotrophic factors (such as nerve growth factor and brain-derived neurotrophic factor), but also neurotoxic factors (e.g., nitric oxide) and proinflammatory cytokines (TNF-*α* and IL-1) [38, 39]. Thus microglial activation is necessary for host defense, but this comes at the prize of additional “bystander damage” [40]. There is evidence that microglia play a detrimental role in various neurodegenerative diseases [41, 42]. However, ample data demonstrate beneficial roles for microglia, for example, by stimulating myelin repair, removal of toxic proteins from the CNS, and the prevention of chronic neurodegeneration [43, 44]. Microglial activation can be caused by neuronal cell death leading to secretion of signaling molecules (including *α*-synuclein, neuromelanin, and active forms of MMP-3) [38, 39, 45, 46]. The expression of MMPs, produced in microglia at sites of inflammation upon activation (such as LPS and Con A [47, 48], could be shown in various studies [1, 49, 50]. Particularly the secreted MMP-2 and MMP-9 [16, 51] seem to be the key modulators (Figure 2).

## 4. MMP-2 and MMP-9 in Inflammation

MMP-2 and MMP-9 are structurally related and share the common feature of a zinc-binding domain. MMP-2 (gelatinase A a 72 kDa type IV collagenase) and MMP-9 (gelatinase B a 92 kDa type IV and type V collagenase) degrade collagens IV and V in their native forms [52]. Besides collagen, MMP-9 targets a variety of other substrates, for example, substance P [53] and b-amyloid (1–40) [54], and MMP-2 cleaves b-amyloid (1–40) and b-amyloid (1–42) [55]. MMP-9 can also degrade human myelin basic protein (MBP), thereby directly contributing to myelin damage [56–58]. 

MMP-9 was called a tuner and amplifier of immune functions [59], because of its assistance in peripheralization of leukocytes in response to chemokines [59] into sites of inflammation and by acting as switch and catalyst at the interplay between the innate and adaptive immune systems. MMP-9 has been implicated in opening the route for immune cells into the neuropil in various diseases, including not only MS but also strokes and brain injuries [1, 25, 31, 60–65]. In fact, the infarct size can be lessened by reducing the MMP-9 activity with a monoclonal antibody [66] or through enzymatic inhibition respectively, gene knockout [65].

**Figure 3:**
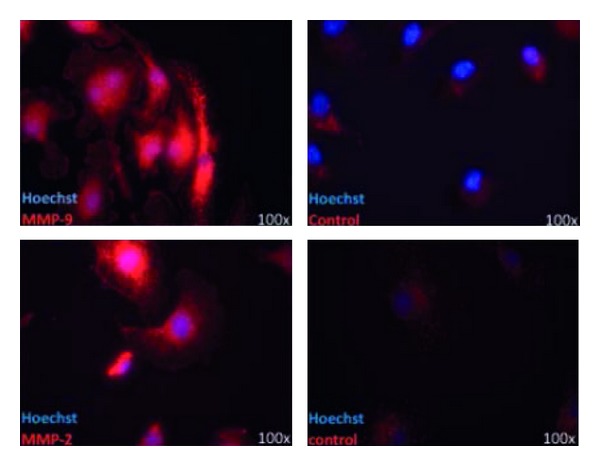
Immunohistochemistry of murine microglia, stained for MMP-2 and MMP-9. Microglia from CD11c GFP mice, fixed with PFA, treated with TBS and NGS, anti-MMP-2 (1 : 125) and anti-MMP-9 (1 : 500); control: BSA and secondary antibodies.

Although the cause of MS remains unknown, MMPs are implicated in the pathology of MS. Focal BBB leakage and extravasation of immune cells into the brain parenchyma are the earliest steps in the pathogenesis of MS as mentioned above [67, 68]. MMPs are effectors of BBB disruption [69]; extensive studies in MS and EAE demonstrated especially activity of MMP-2 [70] and MMP-9 [71, 72]. MMP-9 around blood vessels suggest that MMP-9 might be pathologically involved in the disruption of the parenchymal basement membranes [73], paving the way for infiltrating cells of the immune system [1]. Within the CNS immune cells orchestrate myelin and axonal destruction resulting in severe destruction of normal CNS constituents. The histopathological hallmark of MS is the plaque, a well-demarcated white matter lesion characterized by demyelination and axonal loss. Expression of MMP-1, -2, -3, -7, and -9 in monocytes/macrophages, microglia, astrocytes, and lymphocytes around perivascular cuffs in MS lesions has been described [73–75]. We could also confirm by immunostaining that microglia are sources of MMP-2 and MMP-9 (see Figure 3). 

The secreted MMP-9 can cause demyelination and axonal injury [76, 77]. Axonal damage is considered to be a major cause of secondary progression (with irreversible neurological impairment) [78–80], which seems to be caused not only by T cells [81] but also by microglia/macrophages and their toxic products [75, 82, 83]. Cuzner et al. [84] could confirm enhanced MMP-9 expression in reactive microglia and astrocytes in autopsies from MS brains. Interestingly, intrathecal synthesis of MMP-9 appears to be specific for MS [85, 86]. Around the time of onset of the symptoms in EAE, elevated levels of MMP-9 can be found. The administration of GM6001 (a MMP inhibitor) improved the clinical condition by blocking the BBB injury [87].

The view that MMP-9 is a promoter of neuroinflammation has been additionally supported by the finding that young (3-4 weeks) but not older (7-8 weeks) MMP-9 null mice were less susceptible to development of EAE than wild type controls [71]. In addition, MMP-2 null mice were reported to have an earlier onset and more severe disease compared to wild type controls. Apparently this was related to a compensatory increase of MMP-9 in the MMP-2 null mice [71]. Enzyme inhibitors of MMPs have been shown to ameliorate the clinical course and reduce inflammatory cell infiltration in EAE [87–89]. Treatment of PTx-injected CCL2-overexpressing mice with the synthetic broad-spectrum inhibitor BB-94 (Batimastat) alleviated symptoms of neuroinflammation [90] and put blood-derived cells on hold in perivascular spaces. This was the first evidence that the second step of neuroinflammation, that is, passage of the glia limitans, but not the first, migration across the vascular wall, depends on MMPs. 

The production of MMP-9 is negatively regulated by IL-4 [91], IL-10 [92], and interferon-*β* [93] whereas transforming growth factor-*β* was found to enhance the production of MMP-9 in transformed lymphocytes [94]. Furthermore it was shown that cytokines, chemokines [95, 96], eicosanoides and peptidoglycan, lectins, double-stranded RNS and endotoxin [31, 59, 65, 97, 98] are acting as soluble upregulators [99–101]. Potent stimulators of MMP-9 and MMP-2 expression in cultured astrocytes and microglia are the proinflammatory cytokines interleukin 1 (IL-1), tumor necrosis factor-alpha (TNF-*α*), and lipopolysaccharide (LPS) [102, 103].

Furthermore, interferon-*β* (an immune-modulator that is commonly used in MS) inhibits the expression of MMPs in glial cells. Liuzzi et al. [104] demonstrated that LPS treated microglia secreted higher levels of MMP-9. As soon as the microglia cells were pre-/treated with different doses of IFN-*β* they found dose-dependent inhibition of MMP-9. IFN-*γ* or IFN-*β* was also suggested to inhibit the expression of MMP-9 in human astroglioma and fibrosarcoma cell lines, as well as in primary astrocytes, supposable by the modulation of transcription factors that regulate the MMP-9 transcription [105, 106]. Still an indirect pathway cannot be excluded: IFN-*β* could regulate the MMP expression either through the reduction of proinflammatory cytokines or by the inhibition of the activity of enzymes involved in MMP activation [107]. IFN-*β* also reduces the production of MMP-9 by T cells and monocytes *in vitro* [8, 108, 109] leading to diminished MMP-9 levels in serum of multiple sclerosis patients [110, 111]. This was paralleled by the clinical recovery of the patients, presumably as a result of a significant reduction of T lymphocytes infiltrating in the brain. Besides interferon-*β* also increases gene transcription of TIMP-1, thus attenuating MMP overactivity in MS. Intravenous gamma globulins (IVIG) used in severe cases of MS were shown to diminish the amount of secreted MMP-9 and its mRNA expression [112].

In addition to their detrimental roles MMPs might also have a beneficial effect in MS, as they also have important functions in (the developmental) plasticity of the nervous system [70, 113, 114]: MMP-9 mediates the oligodendrocytes process outgrowth [115]. Cultured oligodendrocytes secrete MMP-9, and cell-associated gelatinases are found at the site of their growing tips of their processes [116]. 

Notably MMP-9 is significantly upregulated in the acute period of spinal cord injury [117, 118] which might promote the maturation of oligodendrocytes and their formation of myelin [119]. MMP-9 [115, 116] and MMP-12 [120] are expressed by oligodendrocytes and seem to be essential for regulating the extension of their processes. Remyelination was impaired in MMP-9 and MMP-9/-12-null mice, correlating with fewer mature oligodendrocytes [121]. Taking that into account the MMP-9 secretion by microglia might allow a microenvironment in lesions for better remyelination and repair [31]. MMP-2 levels increase between 7 and 14 days after spinal cord injury, and MMP-2 null mice do not recover equally well as wild type controls do suggesting that the delayed expression is necessary for ECM remodeling and functional recovery [122]. 

MMP-1, MMP-3, and MMP-8 were also reported to play a role in BBB disruption followed by a leukocyte infiltration into the brain [123, 124]. Woo et al. [125] demonstrated that the mRNA expression of MMP-1, -3, -8, and -9 in primary cultured microglia cells was significantly increased by LPS and other immunostimulants. Furthermore, the inhibition of MMP-3 and MMP-9 could suppress inflammatory reactions in activated microglia (such as iNOS, proinflammatory cytokine expression, and upstream signaling molecules such as MAPKs, which would amplify the inflammatory cascade by initiating the MMP production in an autocrine or paracrine way).

## 5. Conclusion

The data discussed beforehand reinforces the concept that leukocyte transmigration involves distinct molecular mechanisms. MMP-2 and MMP-9 are expressed by microglial cells which contribute to the formation of the glia limitans [126]. These cell types might be responsible for the opening of the glia limitans and the further progression of autoreactive immune cells into the neuropil. Besides the contribution of MMP-9 to BBB breakdown, it is also involved in the generation of autoimmune epitopes as well as the bioavailability of cytokines. 

The treatment of neurological inflammation still remains a challenge today. Targeting MMPs in the CNS may serve as therapeutic option in autoimmune diseases. It is tempting to employ inhibitors of MMP activity to abrogate increased MMP expression (partially driven by microglial activation) within the inflamed CNS. However, MMPs also fulfill beneficial roles in the CNS, including mediation of tissue repair [31], synaptic plasticity [127], learning, and memory [128]. With respect to the multiple roles of MMPs, not only detrimental but also physiological, the need and the judicious application of specific inhibitors against individual MMPs should be highlighted. Due to their bifaced role, timing may also be crucial for therapeutic effects. There is the likelihood that nonspecific MMP inhibitors, although protecting against particular detrimental effects of some MMPs, could block useful actions of MMPs, thus slowing down disease recovery, too. It will be necessary to analyze further therapies aimed at decreasing MMP-2 and MMP-9 expression or activity. 

## 6. Microglia and Gliomas

The most common brain tumors are malignant gliomas, infiltrating diffusely into normal brain parenchyma [129]. So far all current (multimodal) therapeutic approaches were ineffective, and life expectancy from the time of the diagnosis in glioblastoma multiforme is on average 14 months [130–133]. 

In 1921 Rio-Hortega [134], was the first to describe the presence of microglia cells in brain tumors. Microglia contribute substantially (at least 1/3) to the tumor mass of glioblastoma as they make up the largest population of tumor-infiltrating cells [135–138]. Microglia seem to possess a decisive tumor-supporting role by creating a microenvironment, which plays a critical role in tumor initiation and progression [139–144]. This special environment is also an immunosuppressive milieu, where, for example, IL-10 is released [135, 145, 146]. Microglia/macrophages play also an influential role in glioma invasion: there is a positive correlation in their density in gliomas with the invasiveness and grade of gliomas [139, 144]. The antitumor properties, namely glioma-cytotoxic effects of microglia, could only be shown *in vitro* so far [147, 148]. Favoring gliomas' growth could be due to a suppression and/or control of microglial cells by glioma cells and glioma-derived molecules (e.g., their loss of phagocytic function [149]. It was also suggested that, under the influence of glioma cells, microglia develop a different, noninflammatory phenotype suppressing their defense properties [139, 144, 150]. Instead of releasing pro-inflammatory cytokines, microglia upregulate enzymes that facilitate tumor invasion and proliferation. A key mechanism in the expansion and invasion of gliomas is the degradation of extracellular matrix by membrane-bound or secreted proteases such as MMPs [151], especially matrix-metalloproteinase-2 [152] and MMP-9 [151]. 

## 7. MMPs and Glioma Cells

Due to their ECM-degrading ability and confirmed upregulation in almost all cancer entities, MMP-2 has been linked to invasiveness and dissemination [153–155]. Because serum concentration of MMP-2 was significantly elevated in tumor patients, MMP-2 was suggested as a diagnostic and prognostic marker [156, 157]. On the other hand elevated MMP-9 levels in the serum seem to be even more relevant values, because in healthy individuals under physiological conditions MMP-9 is hardly detectable [158]. Abnormal MMP-9 concentrations in patients serum were also shown for brain cancers [159], and notably there is a positive correlation with poor prognosis [160]. 

So far, there is no evidence that links MMP-2 to a special phase of tumor development (in contrast to MMP-9): besides creating a microenvironment in the early phase favoring cancer growth (activation of growth factors), the transition into an undifferentiated phenotype permitting migration and invasiveness is also related to MMP-2 activity, for example, the proteolytic detachment of adhesion molecules like integrins or cadherins or cytoskeleton changes [161, 162]. MMP-2 acts in multiple ways on tumor cells by modulation of their metabolism, their receptor turnover [163], and their resistance to apoptosis [164]. In fact, anti-MMP-2 siRNA-treated glioma cells underwent apoptosis [165] and MMP-2 inhibition autophagy-associated cell death [166]. 

The expression of the MMP-2 gene in human glioma tissues was found to be upregulated in comparison to normal brain tissue, and dramatically increased in glioblastomas [167–169]. MMP-9 expression could be correlated with high malignancy and progression of gliomas [170, 171]. Various studies show that glioma and microglia cells both produce MMP-2 *in vitro* [144, 168] and *in situ* [172]. However, MMP-2 is released as an inactive profrom by glioma cells (especially at the invasive tumor zone), and glioma cells themselves are unable to activate pro-MMP-2. Since the extracellular activator MT1-MMP is highly upregulated in glioma infiltrating microglia [138, 151, 172, 173], glioma cells engage microglial cells to promote their spread and survival [174]. This concept of microglial “abuse” has been impressively demonstrated by the group of Kettenmann [138].

## 8. Pathways of MMP Induction and Suppression in Gliomas

**Figure 4:**
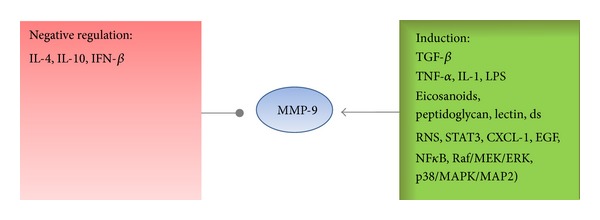
Regulation and induction of MMP-9: a variety of molecules are involved.

Another key player of glioma motility and invasion seems to be FasL, which is expressed in tumor cells. It not only induces apoptosis in T cells thereby leading to local immunosuppression, but blockade of Fas signaling resulted in impaired MMP-2 activity with a subsequent reduction of glioma invasiveness and motility [175]. The expression of MMPs is also facilitated by glioma-derived TGF-*β* which suppresses the expression of TIMPs and also has an immunosuppressive role [176–178]. The inactive precursor of TGF-*β* can be processed by MMP-2 [179, 180], and TGF-*β* induces gene transcprition of MMP-2, thus generating a vicious circle leading to further tumor growth [181, 182] (Figure 4).

Another player in glioma growth, gliomagenesis, and progression is the activation of STAT3 [183]. This signal transducer and activator of transcription protein 3 is constitutively activated in glioblastoma cell lines [184–187] and increases MMP-9 expression and activation in human astrocytoma cell lines [188, 189]. The expression of MMP-2, -9, and -14 in microglia/macrophages was also shown to be enhanced by glioma-derived CX3CL1 (Chemokine (C-X3-C Motif) Ligand) and is significantly associated with the recruitment of microglia into the tumor [190]. Another role in the production of MMP-9 in glioma cells is played by protein kinase C (PKC) [191] and IL-6 is a confirmed growth factor for glioma stem cells, too [192]. The tumor-promoting role of IL-6 may be exerted via MMP-2, whose production is increased by IL-6 [193]. Glioma-induced MMP-2 activity in microglia could be significantly decreased by the A1AR (an adenosine receptor subtype, found on microglia and neurons) [194], which might explain the fact that adenosine treatment leads to decreased extracellular protease activity and thereby exerts its inhibitory effects on glioma invasion. Early studies in MS patients could also show that A1AR activation in microglia interfered with the MMPs production [195].

**Figure 5:**
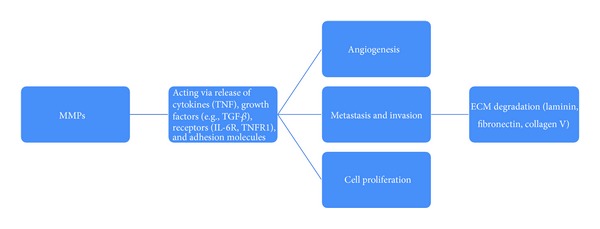
MMP in gliomas: the various roles of MMPs in promoting the growth of cancer cells.

Although the substrate specifity of MMP-2 and MMP-9 overlaps, MMP-9 (in contrast to MMP-2) is highly inducible mostly by integrins, growth factors, and cytokines [13, 196] leading to a defined chronologically and spatially distribution. The expression of MMP-9 is further triggered by autocrine or paracrine mechanisms (IL-1*β*, TNF-*α*, and TGF-*β*), cell binding (to fibronectin or vitronectin), EGF release or distinct molecular pathways (transcription factors NF-*κ*B, Raf/MEK/ERK cascade, or the p38 MAPK/MAPK2-signaling) [180, 196–200] (Figure 5).

In sum, ample data describe the communication between tumor cells and microglia. Microglia and their expression of MMPs could be a crucial target for future therapeutic options in gliomas, due to their profound involvement in tumor progression.

## Figures and Tables

**Table 1:** MMP overview. It was found that MMP-4, -5, and -6 were identical with MMP-2 or -3. Data compiled from Parks et al. 2004 [17] and Sbardella et al. 2012 [160].

MMP group	MMP subgroup	Designation	Alternative name	Matrix substrate	Bioactive substrate
Simple hemopexin-containing MMPs	Collagenases	MMP-1	Collagenase-1/ColA/ColB/Fibroblast collagenase/interstitial collagenase	Unclear: Type I and II fibrillar collagens; collagens I, II, III, VII,VIII, X; gelatin; aggrecan; link protein; entactin; tenascin; perlecan	a2-M; a-PI; a1-antichymotrypsin; IGFBP-2, 3, 5; proIL-1b; CTGF
		MMP-8	Neutrophil collagenase	Collagens I, II, III, V, VII, VIII, X; Fn; entactin; tenascin; gelatin; aggrecan; link protein, Mouse CXCL5	a-PI
		MMP-13	Collagenase-3	Collagens I, II, III, IV, VII, IX, X, XIV; aggrecan; gelatin; Fn; tenascin; osteonectin; Ln; perlecan	CTGF; ProTGF-b; MCP-3; a1- antichymotrypsin; plasminogen
	Stromelysins	MMP-3	Stromelysin-1 (transin-1)	E-cadherin, Laminin, type IV collagen, Latent TGF-*β*1; Aggrecan; decorin; gelatin; Fn; Ln; collagens III, IV, V, IX, X, XI; tenascin; link protein; perlecan; osteonectin; entactin	IGFBP-3; proIL-1b; HB-EGF; CTGF; Ecadherin; a1-antichymotrypsin; a1-PI; a2-M; plasminogen; uPA; pro-MMP-1, 7, 8, 9, 13
		MMP-10	Stromelysin-2	Aggrecan; Ln; Fn; gelatin; collagens III, IV, V, IX, X, XI; tenascin; link protein;	Pro-1, 8, 10
	Others	MMP-12	Macrophage metalloelastase	Latent TNF, Elastin; aggrecan; Fn; collagen IV; gelatin; vitronectin; entactin; osteonectin; Ln; nidogen	Plasminogen; apolipoprotein(a)
		MMP-19	RASI-1	Collagen IV; gelatin; Fn; tenascin; aggrecan; entactin; COMP; Ln; nidogen	IGFBP-3; proIL-1b; HB-EGF; CTGF; E-cadherin; a1-antichymotrypsin; a1-PI; a2-M; plasminogen; uPA; pro-MMP-1, 7, 8, 9, 13
		MMP-20	Enamelysin	Amelogenin; aggrecan; gelatin; COMP	Unknown
		MMP-27	None	Unknown	Unknown

Gelatin-binding MMPs	Gelatinases	MMP-2	Gelatinase A/72kDa gelatinase	CCL-7/CXCL12, gelatin; collagens I, IV, V, VII, X, XI, XIV; Ln; Fn; elastin;aggrecan; osteonectin; link protein	ProTGF-b; FGF receptor I; MCP-3; IGFBP-5; proIL-1b; galectin-3; plasminogen;
		MMP-9	Gelatinase-B	Zona occludens1, *α*1-Antiproteinase, latent TGF-*β*1, latent VEGF, Fibrin, NG2 proteoglycan; gelatin; collagens I, III, IV, V, VII, X, XII; elastin; entactin; aggrecan; Fn; link protein; vitronectin; N-telopeptide of collagen I	ProTGF-b; IL-2 receptor a; Kit-L; IGFBP-3; proIL-1b; ICAM-1; a1-PI; galectin-3; plasminogen

Furin-activated		MMP-11	Stromelysin-3	Fn; Ln; aggrecan; gelatins	a1-PI; a2-M; IGFBP-1
Secreted MMPs		MMP-28	Epilysin	Unknown	Casein

Vitronectin-like insert MMPs		MMP-21	None	Unknown	Unknown

Minimal domain MMPs	Matrilysins	MMP-7	Matrilysin-1 (Pump-1)	Pro-*α*-defensins, FAS ligand, latent TNF, syndecan-1, E-cadherin, Elastin; Aggrecan; gelatin; Fn; Ln; elastin; entactin; collagens, III, IV, V, IX, X, XI; tenascin; decorin; link protein; vitronectin	Both lack the hemopexin-line domain, they process collagen IV but not collagen I, Pro a-defensin; Fas-L; b4 integrin; E-cadherin; proTNF-a; CTGF; HB-EGF; RANKL; IGFBP-3; plasminogen
		MMP-26	Matrilysin-2 (endometase)	Gelatin; collagen IV; Fn; fibrinogen; vitronectin	pro-MMP-9; a1-PI

Type I transmembrane MMPs	MTs-MMPs	MT1-MMP/MMP14		Pro-MMP-2, fibrillar collagens, Fibrin, Syndecan-1, *γ*1-subunit of laminin-5, collagen I, II, III; gelatin; aggrecan; Fn; Ln; fibrin; vitronectin; entactin; proteoglycans; Ln-5	Pro-MMP-2; Pro-MMP-13; CD44; MCP-3; tissue transglutaminase
		MT2-MMP/MMP-15		Fibrin Fn; tenascin; nidogen; aggrecan; entactin; collagen; gelatin; perlecan; Ln; vitronectin	Pro-MMP-2; tissue transglutaminase
		MT3-MMP/MMP-16		Fibrin, Syndecan-1; collagen III; aggrecan; gelatin; Fn; vitronectin.	Pro-MMP-2; tissue transglutaminase
		MT5-MMP/MMP-24		Gelatin; fibronectin; vitronectin; collagen, aggrecan; PG	Pro-MMP-2

GPI-linked MMPs	MTs-MMPs	MT4-MMP/MMP-17		Gelatin; fibrinogen	Unknown
		MT6-MMP/MMP-25		Gelatin; collagen IV; fibrin; Fn; Ln	ProMMP-2

Type II transmembrane MMPs	MTs-MMPs	MMP-23A	Femalysin	Unknown	Unknown
		MMP-23B		Gelatin	Unknown
